# Supplementary material for: Namaste care in the home setting: developing initial realist explanatory theories and uncovering unintended outcomes
Source: BMJ Open. 2020 Jan 22;10(1):e033046. doi: 10.1136/bmjopen-2019-033046 (PMC7045233; doi:10.1136/bmjopen-2019-033046)
Supplement: Supplementary data [file bmjopen-2019-033046supp001.pdf]

Supplementary information 1:

The following criteria are used by the hospice and intended to provide guidance on appropriate referrals for the community based Namaste Care Project.

- The person living with dementia lives at home in the central [location details] or [location details].
- The person living with dementia is most likely in their last year of life.
- The person living with dementia is finding it more difficult to communicate verbally.
- They have become completely dependent on the support of others for activities of daily living.
- They would not now find it easy to leave the house or engage in group activities.
- They would benefit from a gentle, sensory approach, on a one to one basis by a trained volunteer to enhance their wellbeing.
- The person with dementia and/or carer has consented to the referral and is aware that the carer needs to be present in the house during Namaste visits.
